# Consumption of herbal infusions/decoctions and tea in Greece: a Planeterranean perspective on the results of Hydria survey

**DOI:** 10.1186/s12967-023-04781-5

**Published:** 2023-12-11

**Authors:** Ekaterina-Michaela Tomou, Eleni Peppa, Antonia Trichopoulou

**Affiliations:** 1 Research Center for Public Health Research and Education, Academy of Athens, Alexandroupoleos 23, 11528 Athens, Greece; 2 Department of Pharmacognosy & Chemistry of Natural Products, School of Health Sciences, Faculty of Pharmacy, National and Kapodistrian University of Athens, Panepistimiopolis, Zografou, 15771 Athens, Greece; 3 School of Medicine, National and Kapodistrian University of Athens, 75 Mikras Asias Str., 11527 Athens, Greece

**Keywords:** Herbal infusions and decoctions, Herbal teas, Tea, Greece, Green tea, Mountain tea, *Sideritis* spp., *Camellia sinensis*, HYDRIA, Planeterranean

## Abstract

**Background:**

The consumption of various herbal infusions was and is common in the traditional Mediterranean diet, and apparently are used in many other regions of the world outside of the Mediterranean region. The present study aimed to investigate the consumption of herbal infusions/decoctions and tea in a nationally representative sample of Greece, considering also their Planetary use.

**Methods:**

3951 adult participants of the HYDRIA Survey (2013–2014) were included from the 13 regions of Greece. The diet of participants was assessed by two 24-h dietary recalls (24-HDR) and the Food Propensity Questionnaire (FPQ). All food items were analyzed through the HYDRIA Food Composition Tables. Information on socio-demographics, lifestyle, and health characteristics during the baseline was also provided.

**Results:**

26.9% of herbal infusions and decoctions consumers are ≥ 75 years old, pensioners with a low level of education, and residents of urban areas. However, 22.2% of tea consumers belong to the 18–34-year age group, being employed with an intermediate level of education and living in an urban area. Elderly men and women use more herbal infusions/decoctions (55.4%; 40.6%) compared to tea (41.8%; 25.2%). According to FPQ, 7.1% of men and 9.7% of women use herbal infusions and decoctions four times or more per week, while 7.1 and 10.9% of men and women, respectively, consume tea at the same frequency. Green tea, mountain tea (*Sideritis* spp.), and black tea are reported most frequently. In addition, mountain tea (61.2%), black tea (60.3%), mixed herbal infusions/decoctions (58.4%), and sage (59.4%) are consumed mostly at breakfast, while chamomile was reported mainly at dinner or during the night (42.4%).

**Conclusions:**

Based on the FPQ results, the moderate and high consumption of herbal infusions/decoctions and tea is low in Greece, especially among adults (18–64 years). Thus, our results indicate the existence of a generation gap to the attachment to the traditional Med Diet and the consumption of herbal infusions. The present study could encourage future research to focus on herbal infusions and decoctions consumption by other populations for cross-cultural comparison, as well as on emphasizing the value of consuming locally available herbs in a Planeterranean perspective.

**Graphical Abstract:**

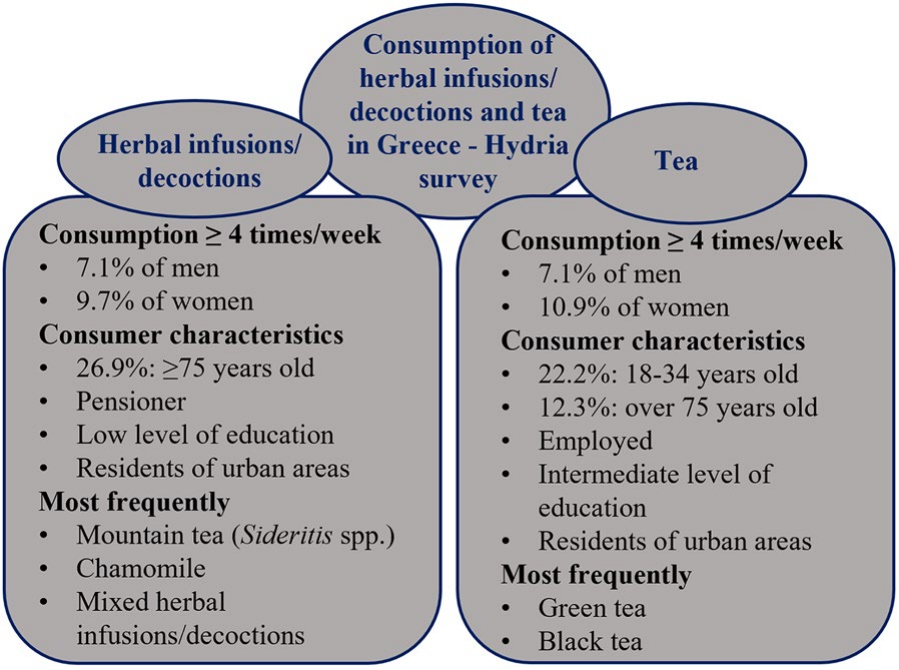

## Background

According to the European Medicines Agency (EMA), “herbal teas” are composed of one or more herbal substances that might have been previously processed (e.g., comminuted, crushed) [1]. These aqueous preparations are directly prepared before use by means of (i) decoction; pouring cold water on the herbal substance(s) and heating until boil (approximately for 15–30 min, if time is not specified), (ii) infusion; pouring boiling water on the herbal substance(s) and allow to soak for 5 to 15 min (if time is not specified), (iii) maceration; soaking the herbal substance(s) in water at room temperature, frequently for 30 min (if time is not specified) [1]. Mixtures of herbal substances (“herbal tea mixtures”) are also prepared to facilitate the mixing of different ingredients [1]. In this paper, emphasis has been given to the herbal infusions and decoctions which from a terminology point of view differentiate from the term “tea”.

Over centuries, herbal infusions and decoctions prepared with herbal substances other than *Camellia sinensis* (L.) Kuntze (Theaceae), have been widely consumed and used in traditional medicines in several countries [2, 3]. In general, they are considered a great source of various nutrients and phytochemicals with beneficial effects on human health [4–9]. Previous studies showed their contribution to lower liver stiffness [10] and to protection against thyroid cancer or other benign thyroid diseases [11]. However, their efficacy and safety data are still limited, and further investigation is essential.

Herbal infusions were very common in the traditional Mediterranean diet, as well as in many other regions of the world outside of the Mediterranean region [12, 13]. The consumption and the preference for specific herbal infusions/decoctions could differ by continent and country, depending on regional culture, traditions, and availability of ingredients. Chamomile (*Matricaria recutita* L.) has a long use as an herbal infusion, being one of the most popular medicinal plants in the world [14–17]. It is considered a traditional herbal medicinal product for the treatment of minor gastrointestinal complaints, symptoms of the common cold, minor ulcers and inflammations of the mouth and throat, irritations of skin and mucosae in the anal and genital region as adjuvant therapy, and minor inflammation of the skin (sunburn), superficial wounds and small boils (furuncles) [18]. Several studies have reported a broad range of pharmacological activity, including antioxidant, anti-inflammatory, gastrointestinal, hypoglycaemic, antidepressant, wound healing, and others [14–17, 19]. The beneficial effects of chamomile could be associated with its bioactive constituents, mainly to its essential oil, and to non-volatile compounds (e.g. phenolic acids, flavonoids, and coumarins) [14–17, 19]. Mountain tea (*Sideritis* spp.; tsai tou vounou in Greek) is mainly consumed in the Greek traditional Mediterranean diet [20], while it is also used by different vernacular names in other countries such as Albania, Bulgaria, Italy, Spain, and Turkey [21–23]. *Sideritis* infusions are recommended as traditional medicines for the relief of mild gastrointestinal disorders and against the common cold [24]. Many studies have investigated their benefits on human health which are attributed mainly to their rich content of polyphenols [23, 25–28]. Lemon balm (*Melissa officinalis* L.) is a medicinal plant that has been widely used in folk medicine around the world, especially in Europe and Iran for the treatment of several diseases [29]. It is considered a traditional herbal medicinal product for the relief of mild symptoms of mental stress and to aid sleep, as well as for the symptomatic treatment of mild gastrointestinal complaints including bloating and flatulence [30]. Previous phytochemical studies on lemon balm have revealed the presence of a wide range of chemical constituents (such as triterpenoids and polyphenols) that could be responsible for its pharmacological effects [19, 31, 32]. In South Africa, several plants are consumed as herbal infusions and decoctions such as rooibos tea [*Aspalathus linearis* (Burm.f.) R.Dahlgren] and honeybush tea (*Cyclopia* spp.) [33–35]. Among them, rooibos tea has gained increasing attention over the years due to its antioxidant activity and associated health-promoting properties [33]. It is reported that 12,500 tonnes of rooibos tea are commonly produced each year in South Africa and 4,500 to 5,000 tonnes are consumed locally, while the rest is exported [36]. It is noteworthy to mention that the term “tea” is generally used in Cape ethnobotany and may refer to both infusions and decoctions. For example, rooibos tea is traditionally prepared as a decoction and not as an infusion [35]. Traditional rooibos tea is prepared through a fermentation process and has a characteristic red-brown colour (“red rooibos”), with a pleasant and slightly sweet flavour [33, 35, 37]. The unfermented product (green in color) is referred to as green rooibos [38]. Although both red and green rooibos are rich in polyphenolic compounds, their concentration differs [38]. Aspalathin, a dihydrochalcone C-glucoside, is a principal component of rooibos tea and its content depends on the extent of fermentation of the plant material and processing conditions [39]. A broad range of herbal teas can be found in China, related to its geographical regions and cultural and/or linguistic groups. For example, Chinese holly tea (*Ilex kaushue* or *Ilex latifolia*), and chrysanthemum tea (*Dendranthema morifolium*), are estimated to have been used for at least 1000 years [40]. India has also a rich history of traditional medicinal systems (like Ayurveda) in which various herbal teas are used [41, 42]. In South America, yerba mate (mate folium; *Ilex paraguariensis* St. Hilaire) is a well-known herbal tea whose production in the three main producing countries (Argentina, Brazil, and Paraguay) is estimated at around 1.4 million tonnes per year [43, 44]. It is also recognised as a traditional herbal medicinal product for symptoms of fatigue and sensation of weakness, as well as to increase the amount of urine to achieve flushing of the urinary tract as an adjuvant in minor urinary complaints [45]. The yerba-mate products are increasingly consumed because of their taste and stimulant properties in various countries of Europe (e.g. Germany, Spain, Italy, France), as well as in the United States, Japan, Korea, and Russia [44].

Tea, made from the leaves of the plant *Camellia sinensis* (L.) Kuntze (Theaceae family), is one of the most universally consumed beverages and is also a part of well-rooted cultural traditions [46–49]. Different tea types could be produced depending on the degree of fermentation, such as green tea, white tea, yellow tea, oolong tea, black tea, and dark tea [46, 50]. These types vary in terms of flavor, color, and composition [51, 52]. Several studies have reported the health benefits of drinking tea, including antioxidant, anti-inflammatory activity, and prevention of chronic diseases (e.g., cancer, metabolic syndrome, cardiovascular disorders, etc.) mainly attributed to their constituents, like polyphenols [53–55]. However, hepatotoxicity risk has been mentioned after the consumption of green tea preparations possibly associated with their catechins content [47, 56].

Herbal infusions and decoctions from various medicinal plants have widely been used in different areas of Greece [57–61]. A recent study reported the use of medicinal and aromatic plants in health disorders related to dietary habits and lifestyle factors in the population of Thrace (NE Greece) [61]. However, there is no information on the consumption of herbal infusions/decoctions and tea in association with the adult Greek population, so far. Therefore, the aim of the present study was to investigate their use in a nationally representative sample of Greece, following the population’s socio-demographic, lifestyle, and health characteristics.

## Methods

### Hydria survey

The diet and the health status of the adult population in Greece were assessed by the cross-sectional HYDRIA survey. It was organized by the Hellenic Health Foundation (HHF) in collaboration with the Hellenic Center for Disease Control and Prevention (HCDCP) of the Hellenic Ministry of Health. The survey was conducted in accordance with the principles in the Helsinki declaration and national laws on the protection of personal data, and it was based on the recommendations of the European Food Safety Authority (EFSA) [62] and the European Health Examination Survey (EHES) [63]. Before enrolling in the study, all participants signed an informed consent form.

### Hydria survey participants

In the HYDRIA survey, a representative sample of the population was taken from the 51 prefectures and 13 regions of Greece, consisting of 4011 men and women who were residents of the country permanently and were at least 18 years old. Details of the HYDRIA survey’s design, methodology, and data collection have previously been described [64].

### Dietary assessment

The diet of participants was assessed by two 24-h dietary recalls (24-HDR) and the Food Propensity Questionnaire (FPQ). For the collection of the 24-HDR data, the HHF Nutrition Tool was used, an automated, interviewer-administered application created by the HHF.

Participants were asked to record every food and beverage they consumed during the previous day. They also had to answer questions regarding types of foods and amounts consumed, as well as preparation practices. The HHF Nutrition Tool contained a food list, facets, and descriptors of food items in line with the LanguaL^™^ Food Description Thesaurus 17 [65] and EFSA’s FoodEx2 classification system. In the HHF Nutrition Tool, validated food photography is included, as well as standard portion sizes for specific food items and household measurements [66]. In addition, among others, there were two facets asking about the origin of the item and the way of cultivation (conventional or organic). A total of 1782 food items were included in the HHF Nutrition Tool food list, and they were grouped into 18 food groups. Its food group was further grouped into other subgroups in accordance with the Langual system and EFSA’s Foodex2 classification system. In the food list, simple items (e.g., tea beverage) were included, which were further specified during the interview with the use of relevant facets and descriptors (e.g., tea beverage, without sugar). Herbal infusions/decoctions and tea were classified as non-alcoholic beverages in separate subcategories. There are approximately 22 items in the herbal infusions/decoctions subcategory (mixed herbal infusions/decoctions, herbal infusions/decoctions not specified, valerian, anise, dittany, spearmint, echinacea, hibiscus, buckthorn, calendula, lemon verbena, marjoram, lemon balm, peppermint, marshmallow, salep, dandelion, linden, mountain tea, nettle, sage, and chamomile). The Hydria participants reported 17 of the previous items (mixed herbal infusions/decoctions, herbal infusions/decoctions not specified, dittany, spearmint, echinacea, hibiscus, buckthorn, lemon verbena, marjoram, lemon balm, peppermint, salep, linden, mountain tea, nettle, sage, chamomile). As for tea, the subcategory contains 8 items (tea beverage, tea not specified, rooibos tea, white tea, black tea, tea decaffeinated, tea with flavors, and green tea) that were all reported by the Hydria participants. All food items were analyzed through the Greek composition table, which was also included in the HHF Nutrition Tool. Furthermore, places where tea and herbal infusions/decoctions could be consumed were recorded, and categorized into seven categories: home, work or school, restaurant or bar or cafeteria, friends’ home, street, traveling, and others.

The FPQ is a short, non-quantitative food frequency questionnaire that records only the frequency of food intake and not the amounts consumed during the previous year. It was used to evaluate the food frequency as well as sporadic food consumption. The FPQ used in the Hydria data collection included questions on 88 food groups or items and 14 dietary supplements. The FPQ was developed and pilot-tested in the context of the PANEU project [67]. Regarding their frequency of intake, participants were asked to select among seven options: (a) never; (b) less than one day per month; (c) one to three days per month; (d) one day per week; (e) two to three days per week; (f) four to five days per week; and (g) six to seven days per week (nearly every day). The questions concerning herbal infusions/decoctions and tea were the following: “How often did you consume herbal infusions and decoctions in the last 12 months?” “How often did you consume tea (e.g., green tea, black tea) in the last 12 months?” For this analysis, the seven categories: (a) never, (b) less than one day per month, (c) one to three days per month, (d) one day per week, (e) two to three days per week, (f) four to five days per week, and (g) six to seven days per week (nearly every day) were classified under four categories: never, low (up to three times per month), moderate (one to three times per week), and high (four times or more per week).

### Socio-demographic, lifestyle, and health characteristics

In the baseline questionnaires, the participants also provided information on socio-demographics and other characteristics. Age was grouped into six categories: 18–34, 35–44, 45–54, 55–64, 65–74, and over 75 years. Education was classified into three categories (low, intermediate, and high). Based on the International Standard Classification of Education, the low category included those who had up to nine years of education or who did not go to school; the intermediate category included those who had completed more than nine and up to twelve years of education; and the high level included those holding a bachelor’s degree or a higher degree. The occupation status of the participants was assessed according to the work reported at the time of the interview and was classified as employed, unemployed, students, pensioners, and those occupied with housekeeping. The residential area of subjects was defined by two variables: the level of residency (i.e. urban, semi-urban, and rural) and the region [namely Attica, Northern Greece (including Epirus, Macedonia, and Thrace), Central Greece (including Peloponnese, Central Greece, Western Greece, Thessaly, and the Ionian islands), and the islands of the Aegean island and Crete]. Waist and hip circumferences were measured during the interview and were used to calculate the waist-to-hip ratio (WHR). The measurements were divided into two groups: no risk and increased risk [68]. Smoking was categorized as a current smoker, a former smoker, or never smoking. Finally, chronic morbidity was assessed by asking the participants whether they had suffered from chronic disease at the time of the interview.

### Statistical methods

The HYDRIA survey used a complex sample design to estimate representative national results on the diet and health of the adult population in Greece. Weighting factors were calculated using the 2011 Greek census, taking into account the study design and the response rate of participants by sex, age group, geographical region, and urbanization level. Weighted percentages of use of herbal infusions/decoctions and tea in men and women were calculated separately and compared among the different socio-demographic, lifestyle, and health variable groups using chi-square tests for survey data. Individuals who did not participate in either of the two 24-HDRs (N = 60) were excluded from the analyses. Thus, the total number of participants in this analysis is 3951. The statistical analysis was performed at 0.05 level of significance, by STATA software (version 13.0; Stata Corp, College Station, Texas, USA).

## Results

### Participants’ socio-demographic, lifestyle, and health characteristics

The participants’ sociο-demographic, lifestyle, and health characteristics were given in Tables 1 and 2. Overall, our results showed that adult men and women consume more tea (68.2; 74.9%) than herbal infusions/decoctions (44.5; 58.4%). Though, elderly men and women use more herbal infusions/decoctions (55.5%; 40.7%) compared to tea (31.8 and 25.2%).

**Table 1:** Socio-demographic, lifestyle, and health characteristics of HYDRIA participants, according to the herbal infusions/decoctions’ consumption

Characteristic	Men			Women			Total		
	Consumers	No consumers (%)	p-value^1^	Consumers	No consumers (%)	p-value^1^	Consumers	No consumers (%)	p-value^1^
	(%)			(%)			(%)		
Age, years									
18–34	6.7	27.1	0.00	16.3	24.1	0.0112	12.1	25.6	0.0000
35–44	13.5	19.5		10.6	18.3		11.9	18.9	
45–54	11.8	18.4		12.0	17.5		12.5	17.9	
55–64	12.5	14.9		19.5	14.6		16.5	14.8	
65–74	19.5	11.1		20.9	11.9		20.2	11.5	
≥ 75	36.0	9.0		19.8	13.6		26.9	11.6	
Education^2^									
Low	53.1	33.3	0.0049	53.3	43.07	0.1371	53.2	38.3	0.0018
Intermediate	27.2	42.4		28.1	36.5		27.7	39.4	
High	19.7	24.3		18.7	20.4		19.1	22.3	
Occupation^3^									
Employed	26.9	54.2	0.0000	26.5	32.3	0.136	26.7	42.9	0.0000
Unemployed	13.4	14.7		11.5	14.9		12.3	14.8	
Student	2.9	5.7		2.9	5.2		2.9	5.4	
Pensioner	56.8	25.4		30.8	21.2		42.3	23.2	
House-keeping	–	–		28.4	26.5		15.9	13.6	
Residential area									
Urban	46.1	60.6	0.0519	57.4	62.2	0.222	52.4	61.4	0.0345
Semi-urban	17.6	15.1		9.8	13.8		13.3	14.5	
Rural area	36.4	24.3		32.7	24		34.3	24.1	
Smoking^4^									
Smoker	20.1	41.1	0.0004	24.8	33.2	0.1443	22.8	37	0.0002
Former smoker	52.0	28.4		13.3	12.8		30.3	20.3	
Never smoker	28.0	30.5		61.9	54.1		46.9	42.7	
WHR^5^									
No risk	12.8	30.0	0.0073	43.3	52.2	0.1004	30.0	41.5	0.0032
Increased risk	87.2	70.0		56.7	47.8		70.0	58.5	
Chronic morbility^6^									
Yes	69.9	49.6	0.0067	76.3	66.2	0.0937	73.5	58.1	0.0007
No	30.1	50.2		23.7	33.7		26.5	41.7	

^1^p-Values comparing herbal infusions/decoctions consumers to herbal infusions/decoctions no consumers estimated by chi-squared test

^2^Low: ≤ 9 years of education; intermediate: 10–12 years of education; high: ≥ 12 years of education including individuals with postgraduate and/or doctoral degrees

^3^Employed category includes unpaid employees in family businesses, paid apprentices, investors, and persons on sick leave, holiday leave, maternity, or parental leave; the student category includes unpaid interns

^4^Smoker category includes current or occasional smokers

^5^Increased risk defined as waist-to-hip ratio WHR ≥ 0·90 in men and WHR ≥ 0·85 in women [68]

^6^Participants reported if they were ill or had been ill the previous 12 months or longer with specific diseases and if diagnosed with the disease by a doctor (self-reported data). The percentages for the category “don’t know” were not included

**Table 2:** Socio-demographic, lifestyle, and health characteristics of HYDRIA participants, according to the tea consumption

Characteristic	Men			Women			Total		
	Consumers	No consumers (%)	p-value^1^	Consumers	No consumers (%)	p-value^1^	Consumers	No consumers (%)	p-value^1^
	(%)			(%)			(%)		
Age, years									
18–34	20.9	25.9	0.2291	23.5	23.2	0.0370	22.2	24.5	0.3776
35–44	21.2	18.9		14.7	18.1		17.5	18.5	
45–54	12.3	18.2		24.6	16.2		19.3	17.2	
55–64	13.8	14.9		12.1	15.4		12.8	15.1	
65–74	16.5	11.3		7.2	13.3		12.4	12.4	
≥ 75	15.3	10.8		18.0	13.8		12.3	12.3	
Education^2^									
Low	30.0	35.1	0.5016	35.7	44.8	0.0654	33.2	40.0	0.1228
Intermediate	42.9	41.7		43.1	35.3		43.0	38.4	
High	27.1	23.2		21.2	19.9		23.8	21.5	
Occupation^3^									
Employed	46.3	52.4	0.1328	29.9	32.1	0.4746	37.1	42.0	0.3970
Unemployed	12.2	14.8		15.9	14.5		14.3	14.6	
Student	6.0	5.4		0.7	4.7		6.6	5.0	
Pensioner	35.1	27.4		23.7	21.9		28.7	24.5	
House-keeping	–	–		28.5	26.9		13.3	13.8	
Residential area									
Urban	56.1	59.4	0.8292	65.9	61.2	0.3496	61.6	60.3	0.7834
Semi-urban	16.5	15.3		10.0	14.0		12.9	14.6	
Rural area	27.4	25.3		24.1	24.9		25.5	25.1	
Smoking^4^									
Smoker	30.0	40.4	0.0578	30.8	32.5	0.8874	30.2	36.4	0.0778
Former smoker	30.3	30.4		12.6	12.8		20.1	21.3	
Never smoker	40.1	29.2		56.6	54.7		49.6	42.3	
WHR^5^									
No risk	30.0	28.8	0.9636	49.9	51.7	0.7133	41.0	40.5	0.8985
Increased risk	71.0	71.2		50.1	48.3		59.0	59.5	
Chronic morbility^6^									
Yes	60.4	50.4	0.1436	66.9	67.3	0.9011	64.1	59.0	0.2261
No	39.6	49.4		33.2	32.7		35.9	40.8	

^1^p-Values comparing tea consumers to tea no consumers estimated by chi-squared test

^2^Low: ≤ 9 years of education; intermediate: 10–12 years of education; high: ≥ 12 years of education including individuals with postgraduate and/or doctoral degrees

^3^Employed category also includes unpaid employees in family businesses, paid apprentices, investors, and persons on sick leave, holiday leave, maternity, or parental leave; the student category includes unpaid interns

^4^Smoker category includes current or occasional smokers

^5^Increased risk defined as waist-to-hip ratio WHR ≥ 0·90 in men and WHR ≥ 0·85 in women [68]

^6^Participants reported if they were ill or had been ill the previous 12 months or longer with specific diseases and if diagnosed with the disease by a doctor (self-reported data). The percentages for the category “don’t know” were not included

About herbal infusions/decoctions, 26.9% of the participants who consume them are over 75 years old with the percentage of men (36%) being higher compared to those of women (19.8%). It was noticed that most of the herbal infusions/ decoctions’ consumers are pensioners with a low level of education, living in urban areas. Furthermore, they never smoke and have an increased risk according to the waist-to-hip ratio (WHR).

Concerning tea consumers, 22.2% belong to the 18 and 34-year age group, whereas 12.3% are above 75 years old. Most of them are employed with an intermediate level of education, living in an urban area. In addition, they are not smokers, and their WHR indicates an increased risk.

### Frequency of consumption based on the FPQ results and the two 24-HDRs

The frequency of consumption of herbal infusions/decoctions and tea according to the answers to the FPQ is shown in Table 3. The herbal infusions/decoctions subcategory includes 17 items (mixed herbal infusions/decoctions, herbal infusions/decoctions not specified, dittany, spearmint, echinacea, hibiscus, buckthorn, lemon verbena, marjoram, lemon balm, peppermint, salep, linden, mountain tea, nettle, sage, chamomile). Similarly, the tea subcategory contains 8 items (tea beverage, tea not specified, rooibos tea, white tea, black tea, tea decaffeinated, tea with flavors, and green tea).

**Table 3:** Frequency of consumption (%) of herbal infusions/decoctions and tea in the HYDRIA survey, by age group and sex, based on the FPQ results

Frequency	Adults (18–64 years)						Elderly (65 + years)						Total (≥ 18 years)					
	Men	Women	p-value	Men	Women	p-value	Men	Women	p-value	Men	Women	p-value	Men	Women	p-value	Men	Women	p-value
	Herbal infusions/decoction (%)			Tea (%)			Herbal infusions/decoction (%)			Tea (%)			Herbal infusions/decoction (%)			Tea (%)		
Never	31.8	29.2	0.0009	40.0	36.0	0.0030	20.2	20.1	0.4261	48.5	48.2	0.9919	29.2	26.7	0.0093	42.0	39.3	0.0558
Low (up to three times per month)	46.1	39.8		33.8	30.2		37.7	39.4		25.2	25.1		44.2	39.7		31.9	28.8	
Moderate (one to three times per week)	17.1	23.3		19.9	22.3		27.9	25.8		16.3	17.5		19.5	24		19.1	21	
High (four times or more per week)	5.1	7.9		6.2	5		14.2	14.7		9.9	9.2		7.1	9.7		7.1	10.9	

Among adult men, 31.8% responded that they never consume herbal infusions/decoctions, and 40% answered that they never drink tea. Though, the corresponding percentages among adult women were 29.2 and 36%, respectively. It was not observed any difference between elderly men and women, who reported no consumption of both herbal infusions/decoctions and tea. In contrast, there was a distinction between elderly men and women (14.2; 14.7%) who consume herbal infusions/decoctions four or more times per week compared with those adult men and women (5.1; 7.9%). In high-frequency tea consumption, the percentages were higher among elderly men and women (9.9; 9.2%) than adult men and women (6.2 and 5%). It is noteworthy to mention that the consumption of herbal infusions/decoctions was statistically significantly higher in adult men compared to adult women (p = 0.0009). The same findings were observed for tea consumption between adult men and women (p = 0.0030), as well as in the total population among men and women (p = 0.0093) for the consumption of herbal infusions/decoctions.

**Fig. 1:**
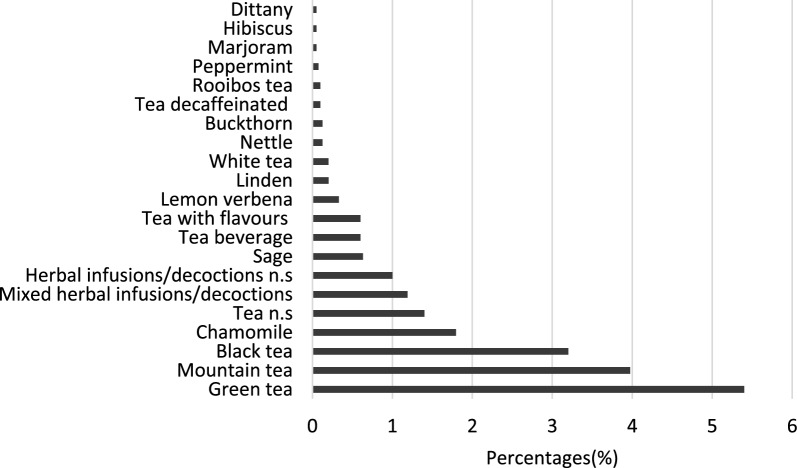
Percentage (%) of HYDRIA participants consuming herbal infusions/decoctions and tea, according to 24-HDR. *n.s:* not specified, *Herbal infusions/decoctions:* mountain tea, chamomile, sage, lemon verbena, lime, linden nettle, buckthorn, peppermint, marjoram, hibiscus, and dittany

Furthermore, the frequency of consumption of herbal infusions/decoctions and tea according to the two 24-HDRs is illustrated in Fig. 1. Among herbal infusions/decoctions, the most popular were mountain tea (4%) and chamomile (1.8%). Green tea (5.4%) and black tea (3.2%) were the most frequently consumed in the subcategory of tea. It should be mentioned that 1% of HYDRIA participants could not specify the kind of herbal infusions/decoctions consumed, while 1.4% of those were unable to identify the tea type.

### Frequency of herbal infusions/decoctions and tea consumption by moments

The frequency and the percentages of herbal infusions/decoctions and tea consumption by respondent-named eating occasions are shown in Table 4. Regarding herbal infusions/decoctions and tea, most respondents consume mountain tea (61.2%), black tea (60.3%), mixed herbal infusions/decoctions (58.4%), and sage (59.4%) at breakfast. Though, chamomile was reported mainly at dinner or during the night (42.4%). On the contrary, green tea is consumed during different moments in the day, at breakfast (38.7%) or during the morning (11.7%), in the afternoon (31%), and at dinner/ night (16.3%). Moreover, HYDRIA participants reported that they consume herbal infusions/decoctions and tea, usually at home (92.4 and 79.7%, respectively). Only 3.3% of herbal infusions/decoctions and 8% of tea consumers used to drink them at work or school, and about 3% at friends’ houses.

**Table 4:** Frequency and percentages of herbal infusions/decoctions and tea consumption by moments

	Breakfast	During morning	Lunch	During afternoon	Dinner/During the night
	N (%)	N (%)	N (%)	N (%)	N (%)
Green tea	116 (38.7)	35 (11.7)	7 (2.3)	93 (31)	49 (16.3)
Mountain tea	126 (61.2)	13 (6.3)	2 (1)	31 (15)	34 (16.5)
Black tea	111 (60.3)	4 (2.2)	5 (2.7)	30 (16.3)	34 (18.5)
Chamomile	33 (38.8)	4 (4.7)	2 (2.4)	10 (11.8)	36 (42.4)
Tea n.s	25 (41.7)	7 (11.7)	1 (1.7)	18 (30)	9 (15)
Mixed herbal infusions/decoctions	35 (58.4)	–	–	13 (21.7)	12 (20)
Herbal infusions/decoctions n.s	23 (48.9)	5 (10.6)	–	5 (10.6)	14 (29.7)
Sage	19 (59.4)	1 (3.1)	–	3 (9.4)	9 (28.1)
Tea beverage	–	6 (21.4)	6 (21.4)	12 (42.9)	4 (14.3)
Tea with flavors	9 (40.9)	2 (9.1)	–	7 (31.8)	4 (18.1)
Lemon verbena	4 (23.5)	4 (23.5)	–	3 (17.6)	6 (35.3)
Linden	3 (37.5)	–	–	1 (12.5)	4 (50)
White tea	3 (37.5)	2 (25)	–	2 (25)	1 (12.5)
Nettle	3 (42.9)	1 (14.3)	–	2 (28.6)	1 (14.3)
Other herbal infusions/decoctions*	9 (39.1)	2 (8.7)	1 (4.3)	4 (17.4)	7 (30.4)
Rooibos tea	1 (16.7)	1 (16.7)	1 (16.7)	1 (16.7)	2 (33.4)
Tea decaffeinated	1 (50)	–	–	–	1 (50)

*n.s:* not specified, *Herbal infusions/decoctions:* mountain tea, chamomile, sage, lemon verbena, linden, and nettle

^*^Other herbal infusions/decoctions: dittany, hibiscus, marjoram, peppermint, and buckthorn, as they were consumed fewer times than the others

## Discussion

This is the first study to investigate the consumption of herbal infusions/decoctions and tea in the adult Greek population and their socio-demographic, lifestyle, and health characteristics. Based on the FPQ results, the moderate and high consumption of herbal infusions/decoctions and tea is low in Greece, especially among adults (18–64 years). It was observed that herbal infusions and decoctions are used at a high frequency in northern Greece. Similar results were noticed by Deligiannidou et al. [61]. However, some differences were observed between our survey and the latter study, including the order of the most consumed herbal infusions/decoctions and tea and the age group of the consumers. These could be attributed to the fact that they focused on a specific area of Greece, as well as the fact that the questionnaire used was online and distributed through social media and word-of-mouth communication. So, the participation of elderly people was limited. In our study, most of the mountain tea and chamomile consumers were over 65 years old and residents of northern and central Greece. Comparing herbal infusions/decoctions non-consumers with consumers in the 18–34 age group, the percentage of people who drink them is significantly lower. This might be attributed to different factors such as (i) the procedure and time needed for herbal infusions/decoctions preparations, (ii) the packaging (usually comminuted herbal substances), (iii) unawareness of the herbal infusions/decoctions health benefits, and (iv) the wide use of coffee by the Greek population. On the contrary, there is not a significant difference between tea consumers and non-tea consumers. It should be mentioned that although the participants in our study were asked about the product origin and the cultivation method during the 24-HDRs, very few of them were able to answer and specify these characteristics.

Landais et al. reported coffee and tea consumption in 10 European countries [69]. Specifically, the average tea consumption was lowest in Greece and Spain, as well as the oldest men tended to drink more tea and coffee than the youngest ones in Greece. It is also mentioned that participants in Greece used to consume their coffee and tea mostly at home, as was also found in our survey.

Previous studies have shown that herbal infusions/decoctions preparation practices play a determinant role in their sensory quality (e.g., flavor, aroma, color), phytochemical content, and beneficial effects. Factors such as brewing/soaking time, heating time, and temperature could affect their bioactive compounds and pharmacological activity, like antioxidant effects [13, 70–76]. In general, consumers prefer convenient and short preparation practices. Rocha et al. (2020) noticed that most of the participants reported taking less than one minute to steep the infusions which is contradictory with the time recommended for the herbal infusions [13]. Therefore, specific guidelines about the preparation practices should be indicated by the producers or on the packaging and be followed by the consumers in order to obtain improved sensory quality, bioactive constituents, and health benefits.

### Herbal infusions/decoctions and tea consumption in Greece compared with other countries

According to the HYDRIA survey, 44.2% of men and 39.7% of women consume herbal infusions/decoctions up to three times per month, followed by 19.5% of men and 24% of women drinking one to three times per week, and 7.1% of men and 9.7% of women use four or more times per week. Furthermore, the participants seem to consume mostly green tea, mountain tea, black tea, chamomile, and mixed herbal teas. Rochas et al. investigated the frequency of consumption in the Portuguese population, reporting that 26% of the participants consume herbal tea up to three times per month, 42% one to three times per week, and 34% five or more times per week [13]. Lemon balm, chamomile, linden, and peppermint are mostly reported by the participants.

Herbal infusions/decoctions and tea consumption differed during the day. Hydria respondents frequently consume mountain tea (61.2%), black tea (60.3%), sage (59.4%), and mixed herbal tea (58.4%) at home most during breakfast, while chamomile is used mainly during the afternoon or dinner or night (54.2%). On the contrary, Polish consumers prefer black, green, and flavored tea mostly at home and between meals (67.3%), as well as during the first breakfast (62.2%) and dinner (48.5%) [77]. The differences in moment consumption during the day depend on the kind of herbal infusions/decoctions and tea and might be associated with their health effects, consumers’ habits/preferences, and the country’s culture.

Comparing studies provided by EFSA with our survey, conducted at the same time (2012–2015) in different countries (Cyprus, Estonia, Finland, France, Latvia, the Netherlands, Portugal, Romania, and Spain) indicates that participants in five of the nine countries used to consume more tea than herbal infusions/decoctions [78]. Furthermore, the percentage of elderly participants who use tea or herbal infusions/decoctions was higher compared to the adult participants. In the HYDRIA survey, in both sexes, elderly herbal infusions/decoctions consumers are more frequent than non-herbal infusions/decoctions consumers, while for tea, there is no difference between tea consumers and no consumers.

### Strengths and limitations of the study

The strengths of the present study are the representative sample of the Greek population over the age of 18 years old, as well as the comparable data collection with those of other European countries, as it follows the recommendations of EFSA [62] and EHES [63]. Furthermore, this study presented some limitations, as participants did not report: (i) the preparation practices of the herbal infusions/decoctions and tea, (ii) the used plant parts (e.g. aerial parts, leaves, etc.), and (iii) the reason for consumption. In addition, our data is relatively older compared to other recent studies that showed the increased herbal infusions/decoctions and tea consumption in several countries as a direct effect of the COVID-19 pandemic [79, 80]. As a result, further studies incorporating recent data should be carried out.

## Conclusions

Herbal infusions are a part of the Mediterranean Diet Pyramid. This study investigated the consumption of herbal infusions/decoctions and tea in the Greek population associated with socio-demographic, lifestyle, and health characteristics. As far as we are aware, no other nationally representative study has reported their consumption in Greece. Overall, the present work revealed that the moderate and high consumption of herbal infusions/decoctions and tea is low in Greece, especially among adults (18–64 years). This observation indicates the existence of a generation gap to the attachment to the traditional Med Diet and the consumption of herbal infusions.

Taking into consideration that Med Diet has been recognized as an Intangible Cultural Heritage of Humanity by UNESCO, we hope to provide important information to the authorities about the value of consuming herbal infusions and decoctions in a Planeterranean perspective that fosters the consumption of locally available herbs with the same properties.

## Data Availability

The data that has been used is confidential.
